# The pathogenesis of cryptoglandular anal fistula: New insight into the immunological profile

**DOI:** 10.1111/codi.16290

**Published:** 2022-08-16

**Authors:** Francesco Litta, Andrea Papait, Donatella Lucchetti, Serafina Farigu, Angelo Parello, Claudio Ricciardi Tenore, Paola Campennì, Antonietta Rosa Silini, Maria Cristina Giustiniani, Ornella Parolini, Alessandro Sgambato, Carlo Ratto

**Affiliations:** ^1^ Proctology Unit Fondazione Policlinico Universitario A. Gemelli IRCCS Rome Italy; ^2^ Dipartimento di Scienze della Vita e Sanità Pubblica Università Cattolica del Sacro Cuore Rome Italy; ^3^ Fondazione Policlinico Universitario ‘Agostino Gemelli’ IRCCS Rome Italy; ^4^ Dipartimento Universitario di Medicina e Chirurgia Traslazionale Università Cattolica del Sacro Cuore Rome Italy; ^5^ Centro di Ricerca E. Menni Fondazione Poliambulanza Brescia Italy; ^6^ UOC di Anatomia Patologica Fondazione Policlinico Universitario A. Gemelli IRCCS Rome Italy

**Keywords:** aetiology, anal fistula, inflammation

## Abstract

**Aim:**

The aetiology of cryptoglandular anal fistula (AF) is poorly understood. Evidence suggests that persistence and/or recurrence of the disease is more related to inflammatory than infectious factors. The aim of this study was to investigate the immune profile of cryptoglandular AF and to perform a histopathological characterization.

**Method:**

Fistulectomy was performed in all patients; healthy ischioanal fat from the same patients was used as a control. Samples were evaluated by the Luminex xMAP system for the detection of 27 analytes. AF tissues were analysed using immunofluorescence. Staining was performed using primary antibodies to identify M1 inflammatory and M2 anti‐inflammatory macrophages. Selective staining of total T lymphocytes and different T lymphocyte subsets was performed.

**Results:**

Twenty patients with AF underwent a fistulectomy. Specific cytokine pathways differentiated AF from healthy tissue: pro‐inflammatory cytokines interleukin (IL)‐1β, IL‐4, IL‐8 and IL‐17 and the anti‐inflammatory cytokine IL‐10 were overexpressed in AF compared with controls. Chemokines involved in macrophage recruitment (CCL2, CCL3, CCL4) were higher in AF than in healthy fatty tissue. Moreover, we showed that Tc17 cells characterize AF patients, thus confirming the enzyme‐linked immunosorbent assay data. Furthermore, elevated infiltration of CD68+ myeloid cells and a reduction of the M1/M2 ratio characterize AF patients.

**Conclusion:**

A combination of inflammatory cytokines, chemokines and growth factors reside in the wound microenvironment of AF patients. For the first time an important prevalence of Tc17 cells and a reduction in the M1/M2 ratio was observed, thus suggesting new insights into the immunological characterization of AF patients.

**What does this paper add to the literature?**

The aetiology of cryptoglandular anal fistula is still poorly understood. This study provides a complete analysis of the immunological microenvironment related to the disease; moreover, for the first time, a specific cell population potentially related to this condition has been described.

## INTRODUCTION

Surgical treatment of anal fistula (AF) is still a debated issue in proctology [1]. Each therapeutic option should have two main objectives: treatment of the fistula and preservation of normal sphincter function [2]. None of the available surgical techniques can be considered as the ‘gold‐standard’ [3]: high success rates are associated with an increased risk of postoperative incontinence, while sphincter‐sparing techniques are often related to a disappointingly high recurrence rate. The pathophysiology of AF is poorly understood, thus partly explaining the persistence of such controversies: therapeutic approaches mainly based on aetiological principles rather than on technical aspects have been advocated [4].

Acute, self‐limiting and resolving inflammation is critically important in the repair process of injured tissues. In contrast, chronic inflammation can lead to excessive tissue damage and deregulated tissue healing. Evidence suggests that epithelialization of the perianal fistula tract and persistence of chronic inflammation may play a decisive role in disease persistence and recurrence [5, 6], while bacterial infection appears to play a role only in the acute phase of perianal sepsis, with no live bacteria being found in the lumen of fistula tracts [7, 8]. However, the presence of bacterial remnants has led to the hypothesis of a role for bacteria in the persistence of AFs [9, 10]. In addition, several studies have reported the presence of pro‐inflammatory cytokines (such as interleukin (IL)‐1β, IL‐8 and IL‐17) [9, 10], that have been reported to contribute to the maintenance or risk of chronicity or recrudescence of the disease [11, 12]. Despite these observations, a precise characterization of the immune infiltrate in terms of T lymphocytes at the site of the anal mucosa of these patients is still lacking. Moreover, in the context of tissue repair, a decisive role is played by macrophages, which are normally classified as M1 or M2 with reference to their pro‐ or anti‐inflammatory activity, mediated by the secretion of different cytokines [13]. Defining the distribution and the phenotype of T lymphocytes as well as macrophages present in the anal mucosa of patients with AF could allow an improved understanding of the immunological profile of these patients and consequently shed light on the altered inflammatory picture found in them. The aim of this study was to investigate the aetiology of AF by characterizing the inflammatory status in terms of cytokine profile and immune cell infiltrate, with a focus on the T lymphocyte subsets. Our findings provide new insight into the immune processes that characterizes this disease.

## METHOD

### Study setting and approval

This was a prospective observational study conducted at the Proctology Unit of the Fondazione Policlinico Universitario A. Gemelli IRCCS, Rome, Italy between May 2020 and April 2021. The local ethics committee approved the study (Prot. ID 2354). All patients signed a written informed consent.

### Patients

A consecutive series of patients referred to our unit and affected by cryptoglandular AF were evaluated by clinical and physical examination (including digital anorectal examination and anoscopy). Moreover, all patients were preoperatively assessed by three‐dimensional endoanal ultrasound (model 2202, BK Medical). A screening endoscopic colorectal evaluation was prescribed according to common guidelines [14]. Inclusion criteria were: cryptoglandular fistula; primary or recurrent fistula, with a single or multiple tracts; medium or high transphincteric fistula (a fistula that crosses at least 30% of the length of the external anal sphincter, assessed by preoperative three‐dimensional endoanal ultrasound); suprasphincteric or extrasphincteric fistula; patient able to provide written informed consent to the surgery; patient with a minimum age of 18 years. Exclusion criteria were: inflammatory bowel diseases; anovaginal or rectovaginal fistula; pouch–anal fistula; active perianal sepsis; patient with a history of radiotherapy involving the anorectal complex; HIV‐infected patient; patient unable or unwilling to provide written informed consent. Continence status was assessed by the Cleveland Clinic Faecal Incontinence score (CCFIS) [15].

This study did not alter the scheduled treatment for the patients, so they were therefore treated according to the usual procedures of our unit.

### Surgical procedure

Two enemas were given before the procedure as bowel preparation, and no antibiotic prophylaxis was prescribed. All patients were treated in the lithotomy position, under general or locoregional anaesthesia; all operations were performed by the senior colorectal surgeon (CR).

The fistula tract and its internal anal opening were explored using a probe and by hydrogen peroxide injection. A complete fistulectomy was performed from the external to the internal fistula opening; then an endorectal flap, closure of the internal opening, sphincter reconstruction or placement of a seton were performed. A macroscopically healthy sample of perianal fat located near the excised fistula was taken from each patient and used as a control.

### Collection of surgical specimens

Immediately after the surgery the collected specimens were quickly frozen in liquid nitrogen and stored at −80°C for subsequent molecular analysis. Part of the excised tissue was also placed in formalin, paraffin embedded and collected for immunohistochemical analysis.

### Multiple cytokine measurement

Tissue samples were thawed, and the washed tissue was cut into small pieces (3 mm × 3 mm) with a knife and homogenized with a mechanical shear homogenizer (IKA‐T10 basic Ultra Turrax, Sigma). The tissue was homogenized in ice and stroked 20 times, pausing for 10–15 s between each homogenization in cell lysis buffer (from The Cell Lysis Kit, #171‐304012, Bio‐Rad) containing protease inhibitor cocktail (#171‐304012, Bio‐Rad) and 3 μl of a stock solution containing 500 mM phenylmethylsulfonyl fluoride (#P‐7626) in dimethyl sulphoxide (#D2650, both from Sigma). The ground tissue was transferred to a clean microcentrifuge tube and frozen at −80°C for 24 h. The next day the samples were thawed, sonicated on ice and centrifuged at 4500*g* for 4 min; supernatants were then collected and stored at −80°C before cytokine analysis. The protein concentration was analysed by Bradford Assay (Bio‐Rad). The multiplex cytokine assay was performed using 500 μg/ml of lysate protein. Multiplex analysis (Bio‐Plex Multiplex Immunoassay System) was performed according to the manufacturer's instructions. IL‐1β, IL‐1RA, IL‐2, IL‐4, IL‐5, IL‐6, IL‐7, IL‐8, IL‐9, IL‐10, IL‐12, IL‐13, IL‐15, IL‐17, eotaxin, fibroblast growth factor (FGF) basic, granulocyte colony‐stimulating factor (CSF), granulocyte monocyte (GM)‐CSF, interferon (IFN)‐γ, IFN‐γ‐induced protein 10, monocyte chemoattractant protein‐1, macrophage inflammatory protein (MIP)‐1α, platelet‐derived growth factor‐BB, MIP‐1β, regulated upon activation, normal T cell expressed and presumably secreted (RANTES), tumour necrosis factor (TNF)α and vascular endothelial growth factor were analysed. Results were analysed using dedicated Bio‐Plex Manager software and are expressed in picograms or nanograms per millilitre as appropriate.

### Histology and immunofluorescence studies

Immunofluorescence studies were performed on sections of mucosal tissue and control samples represented by marginal ischioanal tissues, a region with limited immune cell infiltration and representative of the normal immunological distribution [16].

Paraffin‐embedded sections were dewaxed. Antigen retrieval was performed with HIER Buffer L (1×, pH 6, Thermo Fisher) at 98°C for 20 min. Nonspecific antigen binding was blocked with 10% normal goat serum (Thermo Fisher Scientific).

Immunofluorescence staining was performed to characterize immune cell infiltrates. T lymphocytes were stained with anti‐human CD3 (1:200, A0452, Dako), anti‐IL‐17A (1:200, ab79056) and anti‐CD8a (1:40, ab17147), both purchased from Abcam. For macrophage subset characterization, samples were stained with pan anti‐human CD68 (1:400, M0814, Dako), anti‐human iNOS/NOS II, NT (1:200, ABN26 Millipore) and anti‐human CD163 (1:50, NB110‐59935, Novus Biological) specific antibodies. All incubations with primary antibodies were performed overnight at 4°C.

Secondary staining was performed with secondary antibodies and control isotype: anti‐mouse in horse 594 (DI‐2594), anti‐rabbit in goat 594 (DI‐1594), anti‐mouse in horse 488 (DI‐2488), anti‐rabbit in goat 488 (DI 1488), anti‐mouse in horse CY5 (CY‐2500) and anti‐rabbit in goat CY5 (CY‐1500) all from Vector Laboratories (California, USA). All secondary antibodies were used at a final concentration of 10 μg/ml and were all purchased from Vector Labs. Images were acquired using a Nikon Eclipse Ni‐U microscope at 20× and 40× magnification. Images were processed with ImageJ software (https://imagej.nih.gov/ij) and semi‐quantifications of positive signals from at least three random high‐power fields were analysed, counted and expressed as percentage of total CD3 (for T lymphocytes) or total CD68 (for macrophages). Importantly, T lymphocytes were distinguished by positivity for pan CD3 marker and positive co‐expression of IL‐17A (for CD4+ Th17) or CD8a (for CD8+ T lymphocytes). The resulting cells positive for both IL‐17A and CD8+ were classified as CD8+ Tc17 cells as previously reported [17].

For macrophages, M1 and M2 subgroups were distinguished by differential expression of CD163 typically expressed by M2 macrophages or expression of iNOS, typically expressed by M1 macrophages [18].

### Statistical analysis

Continuous data are reported as mean ± SD (range) and compared using the Mann–Whitney test. Categorical data are presented as frequencies and percentages and compared using the chi‐square test. Evaluation of the cytokine pattern was performed through measurements of the expressions of the considered cytokines (continuous numerical values).

The data representative of the histopathological characterization are shown as violin truncated plots with Tukey variations from at least 20 patients and five controls, from at least three independent experiments. Differences between experimental and control group were analysed by a two‐tailed unpaired Student's *t*‐test using Prism 8 (GraphPad Software). Values of *p* < 0.05 were considered significant. Analyses were performed using SPSS® version 21.0 for Windows® software (SPSS).

## RESULTS

Twenty patients (11 men, mean age 52.1 ± 11.2 years) fulfilled the inclusion criteria and were enrolled in the study. Nine out of 20 patients (45%) were affected by recurrent AF, with the internal fistula opening located anteriorly in eight cases (40%; Table 1). In most cases (16 patients, 80%) the fistula tract was transphincteric. Other patient characteristics are detailed in Table 1. None of the patients included in the study were affected by an immunological disorder; three patients were affected by arterial hypertension and one patient suffered from diabetes mellitus.

**TABLE 1:** Patient characteristics

	Patients, *n* (%)
Mean age (years) (SD)	52.1 (±11.2)
Gender	
Female	9 (45)
Male	11 (55)
Recurrent fistula	
Yes	9 (45)
Not	11 (55)
Location internal opening	
Anterior	8 (40)
Posterior	12 (60)
Parks' classification	
Intersphincteric	4 (20)
Transphincteric	16 (80)
Fistula height	
Low	3 (15)
Medium	13 (65)
High	4 (20)
Surgical treatment	
Seton placement	11 (55)
Internal opening closure	5 (25)
Flap	2 (10)
Sphincter reconstruction	2 (10)

Abbreviation: SD, standard deviation.

All patients underwent a complete fistulectomy, followed by seton placement in 11 cases (55%), closure of the internal fistula opening in five cases (25%) and endorectal advancement flap and primary sphincter reconstruction in two patients (10%). The mean duration of the surgical procedure was 28.4 min (range 19–46 min), with a mean duration of hospital stay of 0.8 day (range 0–1 day). No complications or worsening of continence were registered, so the CCFIS did not change when assessed at the 6‐month follow‐up visit.

### Specific cytokine pathways differentiate AF from healthy tissue

**FIGURE 1:**
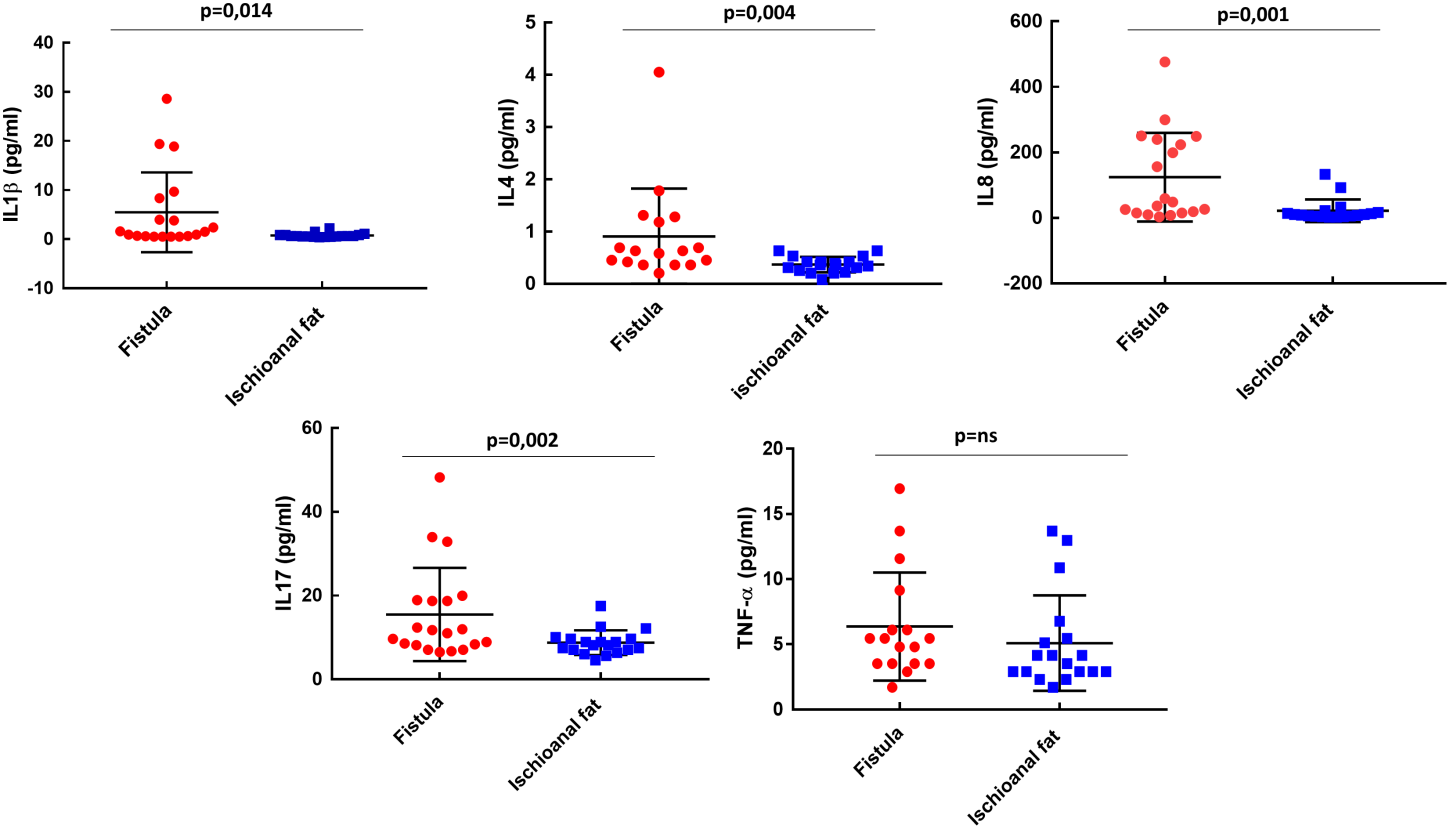
Pro‐inflammatory cytokines interleukin (IL)‐1β, IL‐4, IL‐8 and IL‐17, were all significantly overexpressed in anal fistula when compared with control tissue. Tumour necrosis factor‐α (TNFα) was not overexpressed. In the univariate analysis, levels of the tested analytes were not associated with gender, age, fistula complexity or recurrent disease.

**FIGURE 2:**
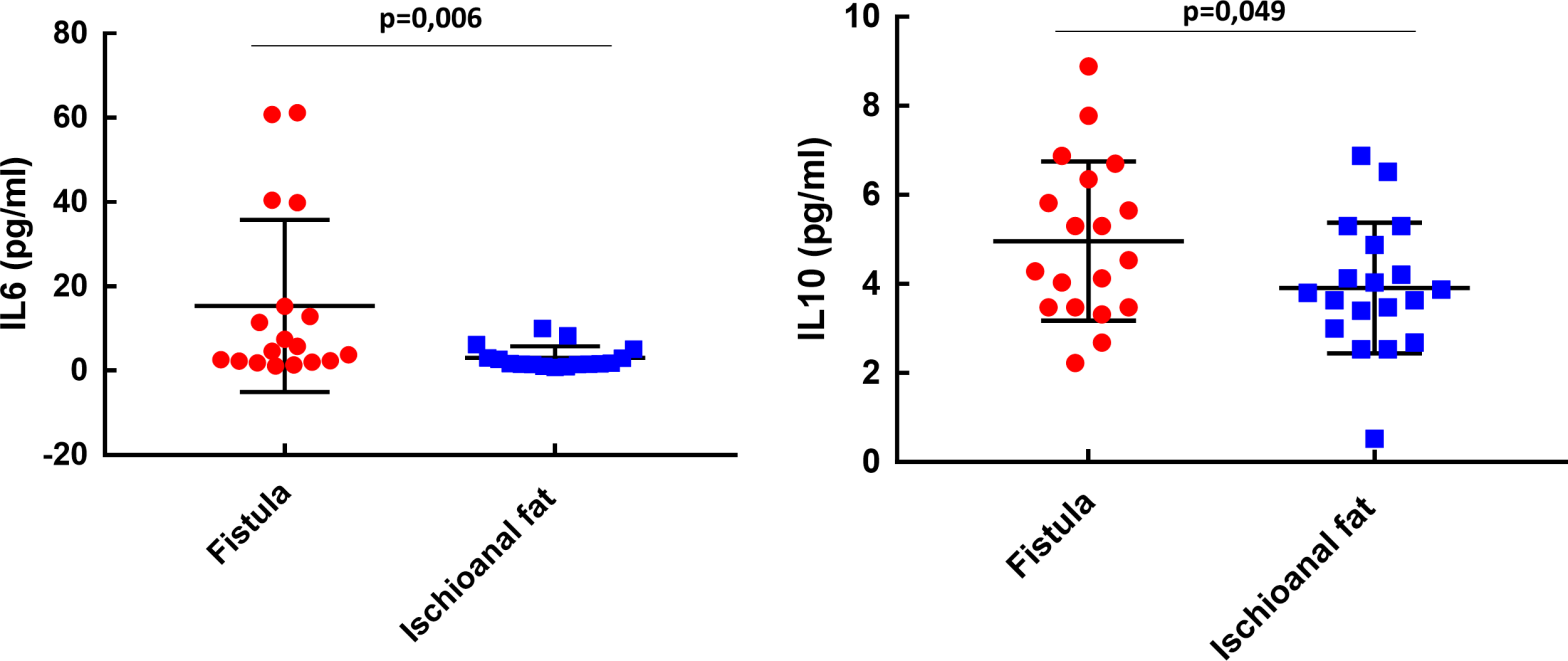
Anti‐inflammatory cytokines interleukin (IL)‐6 and IL‐10 were both significantly overexpressed in anal fistula when compared with control tissue.

**FIGURE 3:**
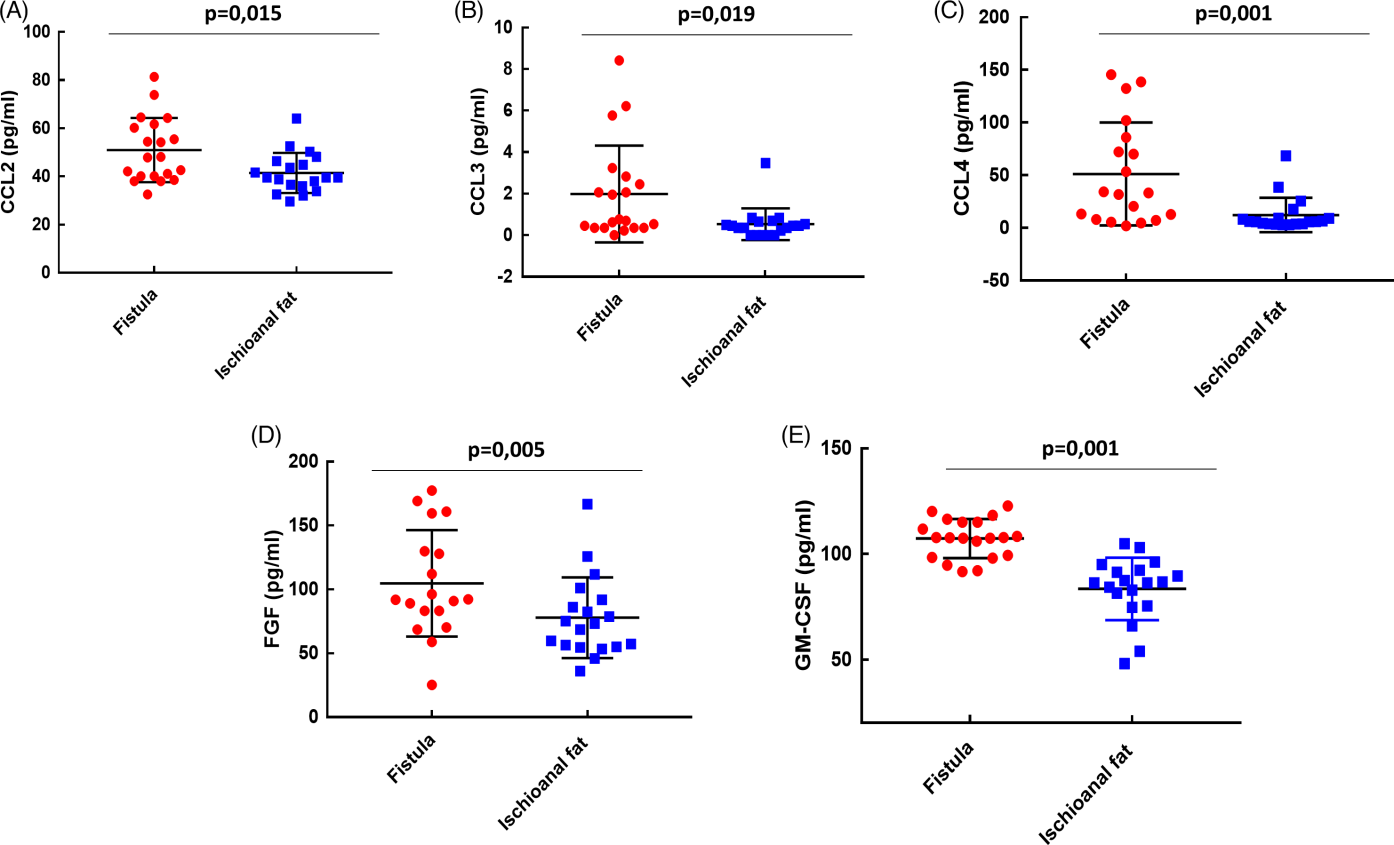
Levels of chemokines involved in macrophage recruitment (A, CCL2; B, CCL3; C, CCL4) and growth factors involved in inflammation (D) fibroblast growth factor (FGF), (E) granulocyte monocyte colony‐stimulating factor (GM‐CSF) were significantly higher in anal fistula than in healthy fat.

The expression patterns of inflammatory and regulatory cytokines in AF tissue and healthy tissue were characterized. Pro‐inflammatory cytokines IL‐1β, IL‐4, IL‐8 and IL‐17 were all significantly overexpressed in AF (*p* = 0.014, 0.004, 0.001 and 0.002, respectively) when compared with control tissue, while TNFα levels were no different (Figure 1). Anti‐inflammatory cytokines IL‐6 and IL‐10 were both significantly overexpressed in AF when compared with control tissue (*p* = 0.006 and 0.049, respectively; Figure 2). Moreover, FGF and GM‐CSF levels were significantly increased in AF when compared with healthy fat as was the level of chemokines involved in macrophage recruitment (CCL2, CCL3, CCL4) (*p* = 0.015, 0.019, 0.001, 0.005 and 0.001, respectively; Figure 3).

### Elevated Tc17 CD8 T lymphocytes characterize AF tissue samples

We performed an in‐depth immunofluorescence characterization and quantification of immune cell infiltrates in AF and compared them with those infiltrated in the control marginal tissue.

**FIGURE 4:**
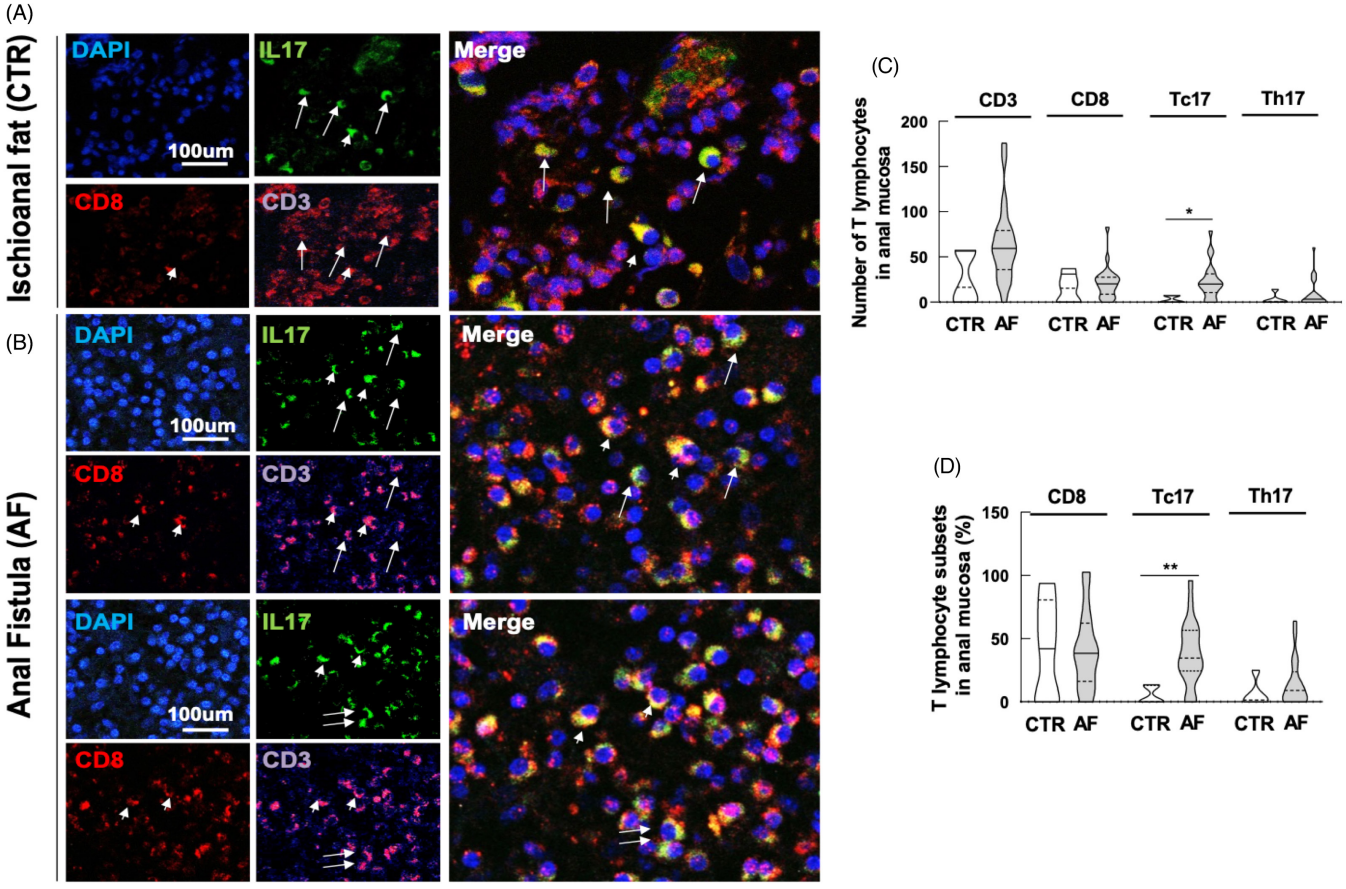
Increased presence of CD8 Tc17 T cells in the inflamed mucosa of patients with anal fistula (AF). The mucosa collected from marginal tissue controls is representative of the normal distribution of immune cells (A) compared with AF marginal zone samples (B). Scale bars: 50 μm. Arrows indicate Th17 cells, short arrows indicate CD8 T lymphocytes, long arrows indicateTc17 CD8 T lymphocytes. (C) Evaluation of the mean number of T lymphocytes counted in three randomly selected high‐power fields (HPFs). (D) Percentage of the different T lymphocyte subsets calculated with regard to the total number of CD3 T lymphocytes counted in three randomly selected HPFs. The data are representative of 20 patients and five controls. *p < 0.05, **p < 0.01.

The analyses of AF tissue showed a greater immune infiltrate (Figure 4B). In fact, we observed an increase in the presence of CD3‐positive T lymphocytes. A closer look into the subpopulations did not reveal any statistically significant differences in the number of CD8 cytotoxic lymphocytes or CD4 Th17 lymphocytes, although a slight increase in the number and percentage of the latter was noted (Figure 4C,D).

Interestingly, we observed an increased presence of CD8 T lymphocytes positive for IL‐17 (or Tc17) expression (Figure 4C,D).

### Elevated infiltration of CD68+ myeloid cells and M2 macrophages characterizes AF tissue samples

**FIGURE 5:**
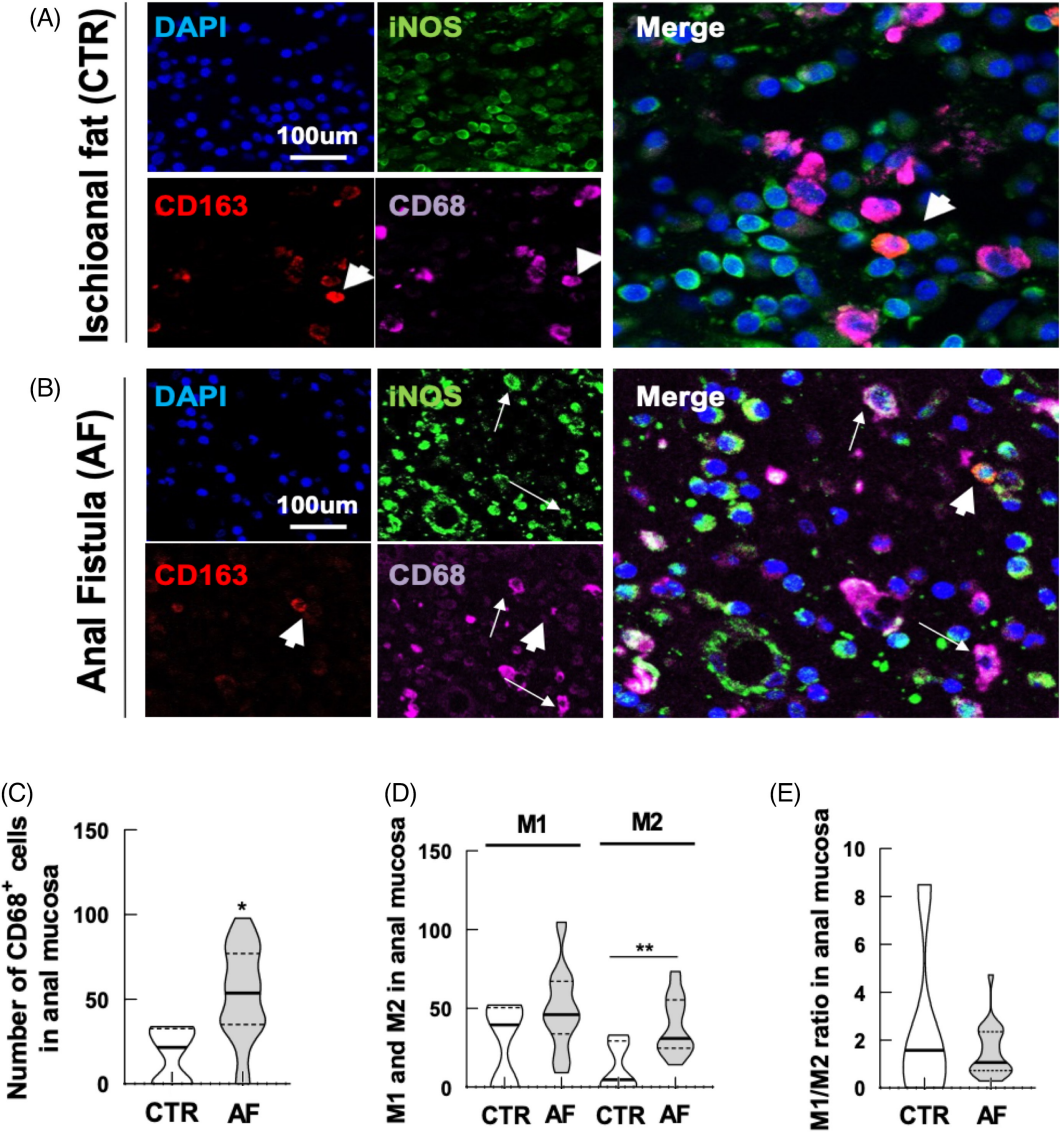
Characterization of M1 and M2 macrophage infiltration in anal fistula (AF) tissue. Immunofluorescence for the pan macrophage marker CD68 and inflammatory marker iNOS for M1 macrophages or CD163 or the M2 anti‐inflammatory macrophages in marginal tissue controls (A) and in AF (B). Long arrows indicate M1 macrophages and short arrows indicate M2 macrophages. Scale bars: 50 μm. (C) Evaluation of the mean number of CD68 infiltrating macrophages counted in three randomly selected high‐power fields (HPFs). (D) Mean number of M1 or M2 macrophages calculated with regard to the total number of CD3 T lymphocytes counted in three randomly selected HPFs. (E) M1/M2 macrophage ratio. The data are representative of 20 patients and five controls. *p < 0.05, **p < 0.01.

In order to investigate the phenotype of macrophages involved in the process of wound healing, and also in light of previously published data indicating an increased expression of IL‐1β in AF patients, we analysed the distribution of M1 and M2 macrophages in AF tissue samples compared with control samples (Figure 5).

We found a strong, statistically significant infiltration of CD68+ myeloid cells (Figure 5C). The distribution of M1 macrophages was analysed by the co‐expression of CD68 and the iNOS marker; M2 macrophages co‐expressed CD68 and CD163 (Figure 5D). In AF samples there was an increase in the polarization/presence of M2 macrophages, resulting in a decreased M1/M2 ratio (Figure 5E).

## DISCUSSION

Surgical treatment of AF is still a debated topic as many aspects are not yet fully known [1]. Most studies published so far have reported the results of several surgical techniques, some even very sophisticated, often with disappointing results [3]. The pathophysiology of the disease is very uncertain, with numerous aspects still unknown: a greater knowledge of the aetiology could lead to new therapeutic strategies, as already advocated [4].

In this study, we report for the first time the prevalence of peculiar CD8 lymphocytes in AF tissues characterized by the expression of the inflammatory cytokine IL‐17, also known as Tc17 [19]. We found that the altered inflammatory state in AF leads to an increased infiltration of CD3 lymphocytes, while no substantial differences were observed in the percentage of Th17 lymphocytes. Furthermore, we report an increased number of CD8 T lymphocytes expressing IL‐17, classified as Tc17 T lymphocytes. To our knowledge, this is the first study to investigate Tc17 in the context of AF. We also observed an increase in the infiltration of CD68+ cells and of M2 macrophages, leading to a reduction in the M1/M2 macrophage ratio.

Normally, Th17 as well as other T lymphocyte subsets are present in the intestinal mucosa to maintain immunity against pathogens and at the same time guarantee a state of tolerance towards commensal bacteria representing the microbiota [20]. In AF patients, an active inflammatory process has been described with a high presence of IL‐1β and IL‐8 [9, 21]. Other studies previously sustained the bacterial hypothesis as a possible mechanism underlying the chronicity of the inflammatory process [7, 22]. Recently, other work has reported that no live bacteria were present in the fistula tract [22], thus dismantling this hypothesis. What is clear is that there is formation of granulation tissue that is constantly present, suggesting a possible role of chronic inflammation [6, 22, 23].

As mentioned earlier, Th17 T lymphocytes play a pivotal role in the rectal mucosa. In fact, these cells play a protective role by promoting the maintenance of the integrity of the mucosal epithelial barrier [24, 25]. Th17 cells are required for the clearance of extracellular bacteria and pathogenic fungi through the secretion of IL‐17 that trigger the activation of other immune cell subsets fostering the clearance of the infiltrating pathogens [24].

CD8 cells have been reported as cells capable of cytotoxic activity by producing IFN‐γ and performing clearance of virus‐infected cells [26]. Recently, CD8 cells have been shown to differentiate into different functional phenotypes, producing cytokines that mirror those secreted by their CD4 lymphocyte counterparts. These subsets have therefore been termed Tc1, Tc2 and Tc17 [27]. Here we report that AF tissue has a significant increase in the number of CD8 Tc17 cells when compared with control tissues. These cells have been reported to produce multiple cytokines, including IL‐2 and TNFα in addition to IL‐17, as well as exhibiting a lower cytotoxic effect than their Tc1 counterparts [28, 29]. These findings are in line with the differences obtained in the total amount of IL‐17 resulting from the Luminex analysis. Furthermore, considering that no significant variations were observed in the amount of Th17 between AF and marginal control tissues, it is conceivable that the observed difference in IL‐17 could be due to the increased presence of the CD8 subset.

A general increase in the frequency of Tc17 has been described in many diseases where the inflammatory response is altered, such as systemic lupus erythematosus and psoriasis [30, 31], as well as in HIV‐negative patients [32]. Another study reported the presence of Tc17 lymphocytes in patients affected by inflammatory bowel disease and suggested that Tc17 cells may impact the altered inflammatory state of patients through the secretion of IL‐17 [33].

Moreover, we observed an increased recruitment of cells positive for the expression of the pan macrophage marker CD68 in AF sections compared with control tissue. This is also reflected in an increased polarization of macrophages towards the M1 inflammatory subset, as highlighted by the increased amount of IL‐1β emerging from the Luminex analysis and confirming our previously published observations [9]. We did not observe significant differences in TNFα levels between the control tissues and AF biopsies, as also previously reported [9]. However, a lack of TNFα variation was also reported in patients with idiopathic and Crohn's‐related AF [34]. This could be explained by the fact that TNFα is produced by M1 macrophages to keep immune defences on pre‐alert [35]. However, TNFα exerts a pro‐inflammatory action, especially in autoimmune diseases such as Crohn's disease, and TNFα has become a major therapeutic target [36]. However, it is noteworthy that in many studies it has been observed that TNFα may also have an anti‐inflammatory action. Indeed, TNFα was reported to promote local steroidogenesis by directly inducing steroidogenic enzymes in intestinal epithelial cells [37].

The increased number of M1 macrophages was balanced by an increase in M2 macrophages, thus resulting in a reduction of the M1/M2 ratio. This was previously reported in other pathologies such as rheumatoid arthritis [38]. The reduction of the M1/M2 ratio was reported in several studies as an indication of a pro‐regenerative process [39, 40], or rather as an attempt, especially in this case, to resolve chronic inflammation [41]. This may also explain the observed increase in IL‐10, which in part may be attributed to the increased infiltration and bias towards M2 macrophages. From a clinical perspective, elevated IL‐10 in patients treated by fistulectomy could be an indication of a pro‐regenerative process even though it is difficult to obtain biopsies from patients who are in the process of recovery.

Indeed, macrophage polarization is a finely regulated process related to resolution of the inflammatory state. In fact, this process occurs even in nonresolving inflammation, since macrophage polarization functions try to restore tissue homeostasis [42, 43].

Limitations of this study are the relatively small sample size, which prevented any subgroup analysis on the clinical outcome, and the absence of an adequate control tissue, which has also been reported by others [9]. As a matter of fact, it is not possible to remove tissue from the intersphincteric space in a healthy patient, nor to use cadaveric tissue because the tissue content would be altered.

## CONCLUSION

A complete analysis of the cytokine pattern characterizing AF patients has been reported. Moreover, our study has identified, for the first time, an important prevalence of CD8 T lymphocytes secreting IL‐17 (Tc17): this finding could shed new light on the identification of potential treatments that could restore the homeostasis of the tissue microenvironment by targeting the CD8 compartment or trying to re‐educate the altered immune system of these patients.

Therefore, further studies will be necessary to explore the mechanisms that regulate the inflammatory response in AF patients, and to establish a causal relationship of the immunological mechanism responsible for the polarization of CD8 T lymphocytes to become Tc17.

## FUNDING INFORMATION

No funding was available for this study.

## CONFLICT OF INTEREST

The authors declare no conflict of interest.

## AUTHOR CONTRIBUTIONS

Study conception: FL, DL, APap, OP, AS, CR. Acquisition of data: SF, APar, CRT, PC, ARS, MCG; Data analysis and interpretation: FL, DL, APap, ARS, MCG, OP, AS, CR; Manuscript drafting: FL, DL, AP; Manuscript revision: SF, APar, CRT, PC, ARS, MCG. Critical revision/supervision: OP, AS; CR; Final approval: FL, DL, APap, SF, APar, CRT, PC, ARS, MCG, OP, AS, CR.

## ETHICS APPROVAL

The study was approved by the local Ethics Committee (Prot. ID 2354).

## PATIENT CONSENT

Patients signed a written informed consent.

## Data Availability

The data that support the findings of this study are available from the corresponding author upon reasonable request.
